# Development and validation of the patient-reported outcome scale for chronic kidney disease

**DOI:** 10.1007/s11255-023-03702-1

**Published:** 2023-07-15

**Authors:** Yu Shi, Shi Pu, Hongmei Peng, Yu Luo

**Affiliations:** 1 School of Nursing, Army Medical University (Third Military Medical University), No. 30 Gaotanyan Street, Shapingba District, Chongqing, 400038 People’s Republic of China; 2 Department of Nephrology, the Key Laboratory for the Prevention and Treatment of Chronic Kidney Disease of Chongqing, Chongqing Clinical Research Center of Kidney and Urology Diseases, Xinqiao Hospital, Army Medical University (Third Military Medical University), Chongqing, 400037 People’s Republic of China

**Keywords:** Chronic kidney disease, Patient-reported outcomes measures, Scale development, Classical test theory, Item response theory, Reliability, Validity

## Abstract

**Purpose:**

The patient-reported outcomes (PROs) measuring patient’s experience and perception of disease are important components of approach to care. However, no tools are available to assess the PROs of chronic kidney disease (CKD). This study aims to develop and verify a PROs scale to evaluate clinical outcomes in CKD patients.

**Methods:**

The theoretical structure model and original item pool were formed through a literature review, patient interviews and references to relevant scales. The Delphi method, classical test theory methods and item response theory method were used to select items and adjust dimensions to form the final scale. Altogether 360 CKD patients were recruited through convenience sampling. CKD-PROs could be evaluated from four aspects, namely reliability, content validity, construct validity, responsibility, and feasibility.

**Results:**

The CKD-PROs scale covers 4 domains, including the physiological, psychological, social, and therapeutic domain, and 12 dimensions, 54 items. The Cronbach’s *α* is 0.939, the split reliability coefficient is 0.945, and the correlation of the scores each item and domain’s coefficients range from 0.413 to 0.669. The results of structure validity, content validity and reactivity showed that the multidimensional measurement of the scale met professional expectations. The recovery rate and effective rate of the scale were over 99%.

**Conclusion:**

The CKD-PROs scale has great reliability, validity, reactivity, acceptability and is capable of being used as one of the evaluation tools for the clinical outcomes of CKD patients.

## Background

The use of patient-reported outcomes (PROs) in clinical care has gained an increasing amount of attention due to patient advocacy and the increasing appreciation of the central role that patients’ symptoms, emotions, and goals play in disease cognition [1–3]. PROs can describe specific symptoms, treatment preferences or aspects of overall health and provide insights into a patient’s well-being that cannot be captured by laboratory data alone [4–7]. PROs are particularly relevant to CKD patients’ care and health, as CKD patients have poorer functional status than those with other chronic conditions; thus, providers are largely unaware of the presence and severity of these symptoms [8, 9]. PROs are being increasingly recognized as a key component of patient-centered kidney disease care.

Chronic kidney disease (CKD) contributes to the global health burden with a high prevalence, poor outcomes, and high cost [10] and is currently the 16th leading cause of years of life lost [11]. It is expected to become the fifth leading cause of death worldwide in the future [12]. CKD progresses to end-stage kidney disease (ESKD). At this point, patients receive renal replacement therapy (RRT), including hemodialysis, peritoneal dialysis, and kidney transplantation [13]. Hypertension, diabetes, and cardiovascular diseases are common comorbidities in patients with ESKD [14]. CKD progression, accompanied by heart failure, fatigue, itching, restless legs, waist muscle soreness, sleep disorders, anxiety, depression, and a series of problems, aggravates the economic, social, physiological, and psychological burden of patients [15]. Historically, the management of patients with CKD was evaluated mainly by clinical results and other hard indicators, such as biological indicators, recurrence rate and mortality, but some symptoms and treatment effects, such as pain, pruritus, and sleep, can only be felt by patients. Capturing and accurately quantifying the subjective feelings of patients is helpful for medical staff to obtain CKD management information on patients and promote clinical decision-making.

Patient-reported outcomes (PROs) come from the status report of the health condition directly collected from the patient. The report generally contains key domains such as symptoms, functional limitations and physical, mental, and social perspectives and can generate a perspective from the patients on the effectiveness of treatment. Patients have become the only source of health outcome endpoint data for many diseases [16, 17]. PROs is often regarded as a vital complement to traditional clinical evidence for studying the treatment impact on patient function and well-being. PROs are useful to discriminate patients and could be a predictor of health conditions, i.e., hospital admissions and health-related quality of life (HRQOL), which is of great significance. In recent years, PROs have been increasingly recognized as valuable instruments for the evaluation of the effectiveness of medical interventions for the HRQOL of CKD patients in many clinical trials [18].

Generic QOL measures have the ability to compare disease burdens between CKD and other conditions; disease-specific measures have better validity, for instance, better responsiveness in some specific conditions [19]. The most commonly used generic QOL tools in CKD include the Short Form-36 Health Survey [20] and its 12-item subset and the Short Form-12 Health Survey [21]. As a disease-specific measurement tool, the Kidney Disease Quality of Life 36-Item Short Form Survey (KDQOL-36) can be used in both CKD-specific and generic QOL domains [22, 23]. The KDQOL-36 augments the Short Form-12 generic core with 24 items and scores 3 kidney-specific scales: Burden of Kidney Disease (4 items), Symptoms/Problems of Kidney Disease (12 items), and Effects of Kidney Disease (8 items). At present, the self-rating scale for CKD patients mainly focuses on symptoms and quality of life without evaluating treatment and social support. PROs is not another word for QOL but involves a wider range of measures than QOL and HRQOL.

In summary, using both classical test theory and item response theory, this study aims to describe the development and validation of the CKD-PROs and to provide guidance for PROs measures. It may also provide a scientific and effective PROs approach to CKD clinical evaluation.

## Methods

### Design and setting

**Fig. 1:**
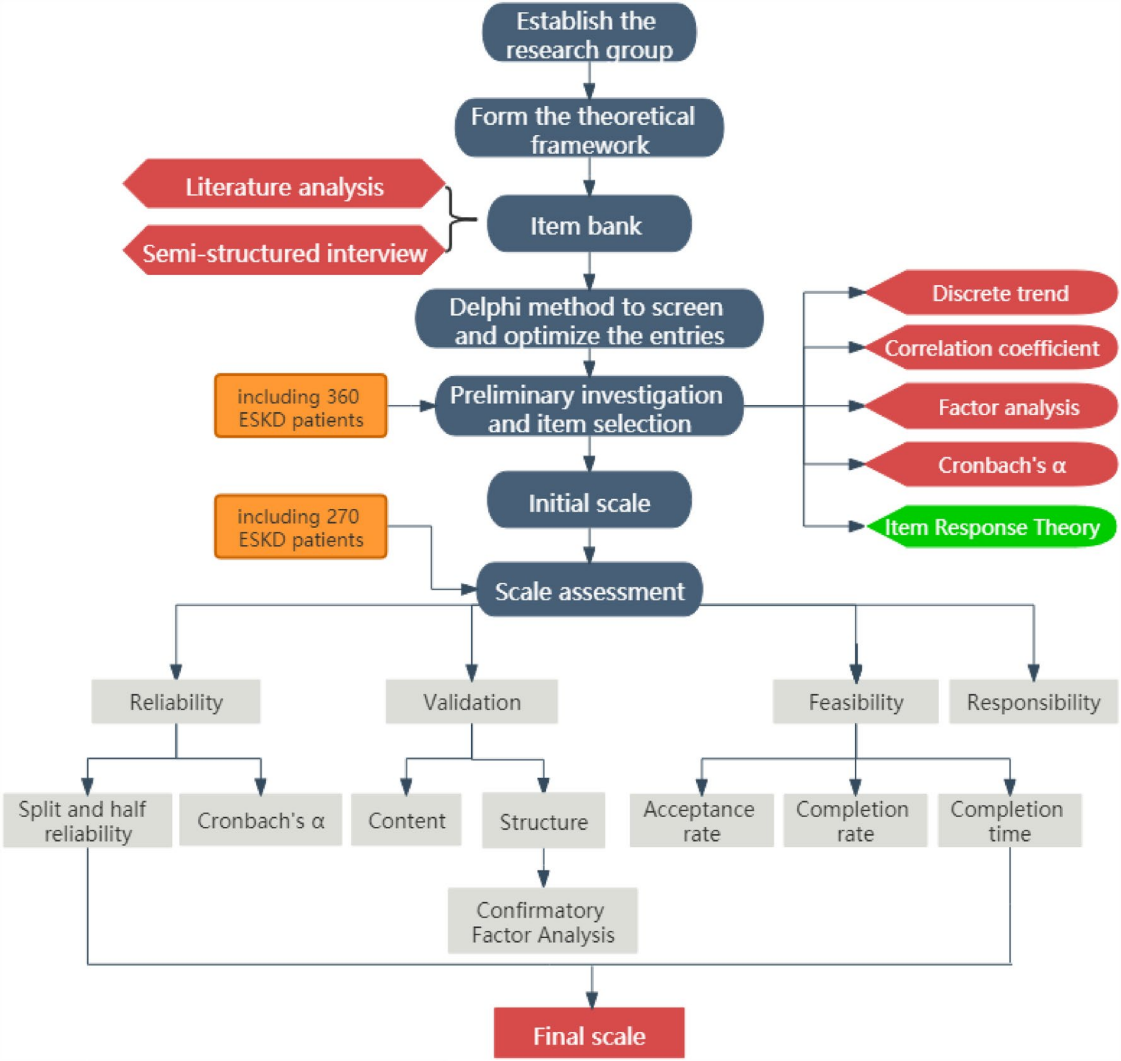
CKD-PROs research roadmap

In this study, a cross-sectional design was used to test psychometric properties. The development and evaluation process was performed in four phases between May 2020 and March 2022. (1) Creation of an item bank: a literature review and patient interviews were performed, and relevant scales were referenced. (2) Formation of the initial scale: the Delphi method was used. (3) Selection of items: Classical test theory (CTT) methods and Item response theory (CRT) method were used to select items and adjust dimensions to form the final scale. (4) Scale validation: the CKD-PROs could be evaluated from the perspectives of reliability, content validity, construct validity, responsibility, and feasibility. Figure 1 shows a flowchart of the developmental process.

### Participants

The study recruited adults with CKD from Southwest China. The development of the scale was conducted in Chinese. The sample size was determined based on the principle of 5 to 10 times the number of items in the scale [24]. A total of 365 paper questionnaires were distributed, out of which 365 were ultimately collected. However, only 360 questionnaires were deemed valid for data analysis. Most of the participants completed the scales independently, but in certain cases, a trained investigator asked questions orally if the participants were unable to complete the task without help. The inclusion criteria were as follows: (1) meeting the diagnostic criteria of chronic kidney disease and glomerular filtration rate (GFR) ≤ 30 ml/(min·1.73 m^2^); (2) age 18–70; (3) patients can understand and complete the scale; and (4) provided informed consent. The exclusion criteria were as follows: (1) patients with acute renal failure due to various reasons; (2) subjects who were unable to complete the questionnaire due to severe organ dysfunction of the heart, liver, or brain; and (3) subjects who did not cooperate with the study due to mental or cognitive disorders. The study was reviewed by the hospital’s Ethics Commission and reached an agreement with all patients by signing the informed consent form.

### Procedure for psychometric property testing

#### Phase I—creation of an item bank

##### Modifying the conceptual framework

**Fig. 2:**
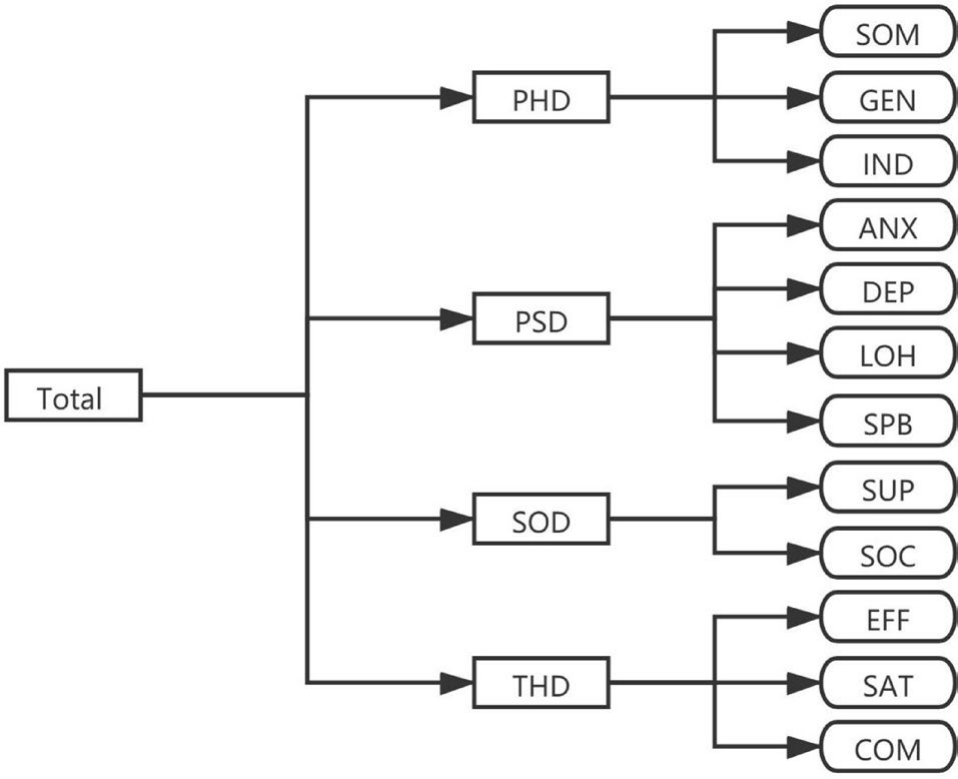
Conceptual framework of the CKD-PROs. *PHD* physiological domain, *PSD* psychological domain, *SOD* social domain, *THD* therapeutic domain, *SOM* somatization, *GEN* General Symptoms, *IND* independence, *ANX* anxiety, *DEP* depression, *LOH* level of hope, *SPB* self-perceived burden, *SUP* Social support, *SOC* Social adaptation, *EFF* effectiveness, *SAT* satisfaction, *COM* compliance

According to the principle and process of making the PROs scale stipulated by The USA Food and Drug Administration (FDA) [25], the existing PROs scale and qualitative research literature for CKD patients were systematically reviewed, the theoretical framework was formed based on the theoretical basis of chronic kidney disease, and the connotation and elements of PROs were classified and analyzed (Fig. 2).

##### Generating items

Under the guidance of the scaling framework, relevant literature related to self-reported outcomes of patients, symptoms, psychology, quality of life, compliance, and satisfaction of patients with CKD were searched for analysis. An objective sampling method was adopted to select 10 patients with CKD for a semistructured interview, and a subject analysis method was adopted to analyze the results. The purpose was to understand the patients’ discomfort symptoms, the impact of the disease, and their expectations for treatment to further enrich the scale item pool.

#### Phase II—formation of the initial scale

In this study, items were preliminarily screened by the Delphi method. Experts were solicited anonymously through several rounds of correspondence until consensus was reached among the panelists for the purpose of forecasting, and the age structure, specialty, and knowledge structure of experts were fully considered in the selection of experts [26]. Eleven experts who had worked for 10 years or more at the general Hospital of Nanjing Military Region, West China Hospital of Sichuan University, Xijing Hospital of Air Force Military Medical University, Xiangya Hospital of Central South University, and another grade-A hospital were selected as the survey subjects (including medical staff at the level of deputy senior medical officer or above). Two rounds of questionnaires were administered in this study, including expert surveys and expert self-evaluations of the basis for judgment, item familiarity, item relevance, and importance, and there was a column for modification suggestions. The Likert 5 score method was used to assign values for the relevance of items. SPSS 22.0 software was used to calculate statistics on the consulting results, arithmetic mean, and coefficient of variation were calculated, and entries were selected by the boundary value method. Items were further modified, deleted, added, or merged after group discussion based on expert opinions. Expert reliability is analyzed from three aspects: expert positive coefficient, expert authority degree and expert opinion coordination degree [27].

Thirty patients were selected for a pilot survey to ascertain numerous variables: whether patients could understand the items, how to answer the items, and whether their understanding of the items was the same as the contents of the scale or was there a need to modify or delete items that were difficult to understand; a cultural debugging of the form and content of the scale was also conducted. In the end, no entries were modified or deleted.

#### Phase III—selection of items

##### Classical test theory (CTT)

According to the methods and principles of entry screening stipulated by the WHO [25], the following four-item screening statistical methods are adopted in this project for screening: (1) discrete trend: the standard deviation (SD) of each item’s score is used to measure the discrete trend; it is recommended to delete items with an SD < 0.7 [28]; (2) correlation coefficient: according to the expected theoretical structure, the correlation coefficient between each item and the total score is calculated, and the items with *r* < 0.4 will be deleted [29]; (3) factor analysis: factor analysis and orthogonal rotation with maximum variance are performed. In this study, items with a loading < 0.4 on each factor and indexes with similar load coefficients in ≥ 2 factors without specificity were deleted. (4) Cronbach’s *α* coefficient: if Cronbach’s *α* coefficient increases greatly after removing a single item, it indicates that the existence of this item has an impact on reducing the internal consistency of this aspect; thus, it should be deleted.

##### Item response theory (CRT)

This method uses probability to explain the relationship between subjects’ responses to items and their potential ability traits [30]. The Likert 5-point scoring method was adopted in this study, so the Samejima rank response model was adopted to estimate the distinction parameter (a) and difficulty parameter (b) of each item. An item with a distinction of *a* < 0.4 should be excluded. Parameters b1, b2, b3, and b4 correspond to four difficulty levels, where b1 is the category threshold between option 1 and option 2, and so on, and b1 < b2 < b3 < b4. The difficulty level parameter generally ranges from − 3 to 3 [31].

#### Phase IV—scale validation

##### Reliability analysis

Reliability refers to the consistency of measurement results. (1) Split reliability: the split reliability method divides all variables into two halves and calculates the correlation between the two parts. In this study, items are arranged according to the classification of items and divided into half in the order of odd and even, usually ≥ 0.7. (2) Cronbach’s *α* coefficient reflects the average correlation between variables and can estimate the scale and the internal consistency of each field; values above 0.7 are considered acceptable [32].

##### Validity analysis

The evaluation is about bias or what proportion of systematic error is included in the measurement results. (1) Structural validity: confirmatory factor analysis (CFA) was used to build a measurement model between indicator items and their dimensions. Relatively reliable indicators include the nonnormed fit index (NNFI), comparative fit index (CFI), adjusted goodness-of-fit index (AGFI) and root mean square of the approximate error (RMSEA) [33]. (2) Content validity refers to the extent to which a particular item reflects a content category. In this study, the content validity index (CVI) was used for quantitative analysis. The project was retained if the CVI was more than 80%.

##### Dimensional correlation

Item correlation refers to the degree of relevance between an item and its domain. When the correlation coefficient *r* is > 0.4, the dimensional correlation is considered acceptable.

##### Response analysis

Response analysis refers to the ability to detect the minimum changes in patients’ quality of life. The scores and total scores of each field measurement before and after treatment were calculated and statistically analyzed. The two‐sample *t* test was used as the statistical method, and *p* < 0.05 was regarded as the indication that the scale has the capability to discriminate the control group from the CRF group.

##### Feasibility evaluation

Feasibility evaluation mainly reflects the acceptability of the questionnaire. Common indicators include the scale recovery rate, efficiency rate, and time to complete each scale.

##### Data analysis software

LISREL version 8.8 of Confirmatory factor analysis (CFA) was used in this study, and MULTILOG version 7.0 of IRT analysis was performed. Other data analysis was processed by SPSS version 22.0. If data from individual items are missing, item scores were replaced based on the average data. If at least three methods passed the filter, it was selected as the final item.

## Results

### Participants’ characteristics

During the item filtering process, we conducted a clinical survey of 365 patients, and 360 valid samples were collected. The patients’ average age was 54 ± 12.56, and there were 187 males (51.9%) and 173 females (48.1%). To examine the reliability and construct validity of the scale, 272 patients were surveyed with the final scale. The mean age of these patients was 51 ± 13.48 years; 188 were males (69.1%), and 84 were females (31.9%). 47 (13.1%) individuals had primary school education or below, 236 (65.5%) had junior high or senior high school education, and 77 (21.4%) had undergraduate education or above; 271 (75.3%) were married, and the remaining 89 (24.7%) were single, including those who were divorced; 238 (66.1%) of the patients were engaged in paid work, and the remaining 122 (33.9%) were unemployed; 21 (5.8%) individuals had high income (annual household income > 150,000 CNY), 192 (53.3%) had moderate income (annual household income between 50,000 and 150,000 CNY), and 147(40.9%) had low income (annual household income < 50,000 CNY); 58 (16.1%) cases had concurrent diabetes, 125 (34.7%) had concurrent hypertension, 43 (11.9%) had concurrent cardiovascular disease, and 66 (18.3%) were infected with hepatitis B. The distribution of personal and clinical characteristics of the study patients is shown in Table 1.

**Table 1:** Demographic and disease characteristics of patients

Characteristics	Date	
	Item selection (*n* = 360)	Validation (*n* = 270)
Age (years) mean ± SD	54 ± 12.56	51 ± 13.48
Gender *n* (%)		
Female	173 (48.1%)	84 (31.9%)
Male	187 (51.9%)	188 (69.1%)
Educational level *n* (%)		
Primary school and below	47 (13.1%)	51 (18.7%)
Secondary and high school	236 (65.5%)	171 (62.9%)
Bachelor degree or above	77 (21.4%)	50 (18.3%)
Marital status *n* (%)		
Married	271 (75.3%)	221 (81.3%)
Single	89 (24.7%)	51 (18.7%)
Employment status *n* (%)		
Employed	238 (66.1%)	184 (67.6%)
Unemployed	122 (33.9%)	88 (32.4%)
Income status *n* (%)		
High	21 (5.8%)	32 (11.8%)
Average	192 (53.3%)	153 (56.3%)
Low	147 (40.9%)	87 (31.9%)
Stage		
CKD1-3	263 (73.1%)	176 (64.7%)
CKD4-5	97 (26.9%)	96 (35.3%)
Additional illness *n* (%)		
Diabetes		
Present	58 (16.1%)	21 (7.7%)
Absent	302 (83.9%)	251 (92.3%)
Hypertension		
Present	125 (34.7%)	111 (40.8%)
Absent	235 (65.3%)	161 (%59.2)
Heart disease		
Present	43 (11.9%)	23 (8.5%)
Absent	317 (88.1%)	249 (91.5%)
Hepatitis B		
Present	66 (18.3%)	72 (26.5%)
Absent	294 (81.7%)	200 (73.5%)

### Psychometric properties of the level of CKD-PRO

#### Item generation and selection

In total, 79 entries were generated through literature analysis and patient interviews. In addition, in the physiological, psychological, social, and therapeutic domains, there were 27, 20, 12 and 20 items, respectively.

Subsequently, a total of 22 questionnaires were distributed in the 2 rounds of this study. The recovery rates of expert consultation questionnaires in the first and second rounds were 100% and 90.9%, and the positive coefficients of experts were 100% and 90.9%, respectively, indicating a high degree of participation and importance in this study. The Kendall coordination coefficient *W* of the second round of consultation was 0.254, which was statistically significant by the *χ*^2^ test (*χ*^2^ = 175.500, *p* < 0.001). The coordination coefficient of each dimension was between 0.201 and 0.273 (*p* < 0.05), indicating that the expert scores were consistent. The coefficient of variation for the importance of each item was 0–0.34, indicating that the experts agreed on the content of the index. The coefficient Cr value of expert authority degree was 0.92, indicating high reliability of expert scoring and authoritative and reliable research results.

In the first round of expert consultation, 9 items—dry mouth, constipation, leg discomfort, tinnitus, slow reaction, bad emotional control, stable blood pressure, protein intake control and water intake control—were deleted due to weak correlation, repeated content, and inconsistent fields. Four items—foam urine, skin damage, folk prescription purchase, blood pressure and blood sugar monitoring—were added, and some items were revised and improved. In the second round of expert consultation, six items were deleted: soreness and pain in the back, memory loss, confidence in the future, financial burden, social status, and impact on daily work. After 2 rounds of expert consultation and discussion and modification by the research group, a preliminary scale containing 64 items in 12 dimensions was formed.

Finally, researchers analyzed the data from 360 patients with CKD. The discrete trend method, correlation coefficient method, factor analysis method, Cronbach’s *α* coefficient method and item response theory were used to screen the scale items, and the items were removed with strict standards. The items that were recommended to be retained by at least three methods were selected, that is, the items that did not meet the standards by more than two methods were deleted. The final scale consists of 54 items, which belong to 12 dimensions and 4 domains. Among them, 16 are in the field of physiology, 14 in the field of psychology, 9 in the field of society and 15 in the field of therapy. The results are shown in Table 2.

**Table 2:** Results of the item-selection phase using CTT and IRT

Item	SD	Correlation coefficient	Factor loading	Alpha	IRT					Outcome
					a	b1	b2	b3	b4	
PHD1	0.984	0.499	0.544	0.867	0.61	− 2.09	− 1.07	− 1.35	− 0.85	√
PHD2	0.768	0.417	0.625	0.867	0.75	− 3.53	− 2.01	− 1.75	0.40	√
PHD3	0.591	0.358	0.543	0.866	1.01	− 3.81	− 3.01	− 2.72	− 0.68	×
PHD4	1.004	0.480	0.708	0.869	0.46	− 1.02	− 0.92	− 0.27	0.32	√
PHD5	0.857	0.511	0.656	0.867	0.85	− 2.52	− 1.22	− 1.37	0.33	√
PHD6	1.442	0.438	0.616	0.869	0.34	− 2.91	− 1.45	− 0.61	3.06	√
PHD7	0.869	0.524	0.687	0.868	0.58	− 2.66	− 1.38	− 0.30	0.28	√
PHD8	0.687	0.387	0.713	0.866	0.88	− 3.84	− 3.07	− 2.37	− 0.21	×
PHD9	0.601	0.258	0.458	0.867	0.72	− 8.79	− 5.34	− 4.80	− 0.35	×
PHD10	0.595	0.263	0.538	0.867	0.75	− 8.55	− 4.94	− 4.61	− 0.84	×
PHD11	0.827	0.462	0.627	0.867	0.60	− 1.07	− 0.37	− 0.14	0.37	√
PHD12	0.95	0.405	0.749	0.868	0.48	− 7.83	− 4.71	− 3.98	− 0.04	√
PHD13	1.062	0.590	0.586	0.867	0.55	− 5.59	− 3.64	− 3.07	0.03	√
PHD14	0.954	0.612	0.668	0.865	0.87	− 4.38	− 2.33	− 1.53	1.33	√
PHD15	0.818	0.473	0.480	0.866	0.95	− 4.06	− 3.22	− 2.63	− 0.66	√
PHD16	0.881	0.255	0.394	0.867	0.55	− 7.61	− 4.41	− 3.81	− 0.23	×
PHD17	0.923	0.372	0.636	0.867	0.54	− 7.12	− 4.59	− 3.41	− 0.65	√
PHD18	0.765	0.480	0.562	0.868	0.52	− 8.17	− 5.93	− 4.43	− 0.57	√
PHD19	0.439	0.226	0.621	0.868	0.94	− 7.61	− 4.87	− 4.36	− 1.83	×
PHD20	1.411	0.501	0.683	0.863	1.00	− 1.71	− 0.35	0.22	1.63	√
PHD21	1.328	0.585	0.616	0.868	0.59	− 3.78	− 2.30	− 2.00	− 0.21	√
PHD22	1.217	0.484	0.729	0.866	0.80	− 3.16	− 1.86	− 1.42	0.69	√
PSD1	1.359	0.459	0.601	0.866	0.64	− 2.54	− 0.85	− 0.03	2.71	√
PSD2	1.277	0.415	0.454	0.869	0.35	− 4.98	− 1.75	1.22	5.13	√
PSD3	0.914	0.458	0.615	0.865	1.30	− 2.78	− 2.09	− 1.78	− 0.42	√
PSD4	1.051	0.402	0.509	0.865	0.77	− 4.13	− 2.43	− 1.91	0.83	√
PSD5	1.115	0.490	0.684	0.864	1.15	− 2.57	− 1.42	− 0.91	0.99	√
PSD6	1.167	0.529	0.661	0.870	0.24	− 4.67	3.16	5.53	11.65	√
PSD7	1.369	0.475	0.714	0.864	1.08	− 1.79	− 0.43	0.14	1.55	√
PSD8	1.327	0.508	0.649	0.863	1.38	− 1.64	− 1.06	− 0.41	0.65	√
PSD9	0.633	0.356	0.573	0.866	1.02	− 4.67	− 3.97	− 3.55	− 0.48	×
PSD10	0.786	0.486	0.757	0.866	1.21	− 3.74	− 3.26	− 1.36	0.72	√
PSD11	0.713	0.423	0.647	0.866	1.26	− 4.72	− 3.84	− 1.47	0.66	√
PSD12	0.786	0.479	0.616	0.866	1.24	− 3.89	− 2.99	− 0.93	1.26	√
PSD13	0.982	0.502	0.577	0.867	0.82	− 4.50	− 2.84	− 2.09	− 0.25	√
PSD14	1.239	0.405	0.707	0.865	0.96	− 2.77	− 1.54	− 0.91	0.51	√
PSD15	1.167	0.507	0.798	0.864	1.38	− 2.01	− 1.45	− 1.09	0.27	√
SOD1	1.406	0.441	0.440	0.865	1.01	− 1.38	− 0.37	0.31	1.90	√
SOD2	0.981	0.408	0.585	0.865	1.09	− 3.45	− 2.21	− 1.51	− 0.01	√
SOD3	1.324	0.471	0.749	0.866	0.84	− 2.61	− 1.05	− 0.50	1.39	√
SOD4	1.242	0.511	0.624	0.869	0.27	− 3.90	2.74	4.55	9.14	√
SOD5	1.17	0.341	0.556	0.866	0.78	− 3.59	− 2.34	− 1.12	0.53	√
SOD6	1.286	0.379	0.477	0.866	0.77	− 2.81	− 1.24	− 0.20	1.74	√
SOD7	1.417	0.620	0.678	0.869	0.39	− 4.15	− 0.91	0.05	3.44	√
SOD8	1.212	0.623	0.540	0.867	0.64	− 3.91	− 2.47	− 1.08	1.03	√
SOD9	1.328	0.791	0.407	0.864	1.15	− 1.80	− 0.69	0.25	1.42	√
THD1	1.024	0.394	0.809	0.867	0.60	− 2.62	0.42	2.75	6.61	√
THD2	1.038	0.512	0.834	0.867	0.70	− 2.87	− 0.24	1.76	5.29	√
THD3	0.813	0.533	0.678	0.866	0.88	− 5.04	− 3.57	− 2.45	− 0.67	√
THD4	0.766	0.660	0.704	0.867	0.64	− 6.45	− 4.80	− 4.13	− 1.47	√
THD5	1.338	0.476	0.486	0.868	0.52	− 1.58	0.26	2.06	4.63	√
THD6	0.838	0.418	0.695	0.865	1.16	− 3.41	− 2.30	− 0.12	2.23	√
THD7	0.701	0.372	0.718	0.866	1.16	− 4.51	− 3.70	− 1.37	1.35	×
THD8	0.963	0.496	0.660	0.865	1.14	− 3.01	− 1.59	0.06	2.14	√
THD9	0.868	0.541	0.666	0.864	1.65	− 2.76	− 1.43	0.11	1.88	√
THD10	0.818	0.385	0.560	0.866	1.13	− 4.24	− 2.76	− 1.83	0.04	√
THD11	0.627	0.324	0.59	0.867	1.15	− 4.16	− 3.41	− 2.92	− 0.99	×
THD12	0.936	0.519	0.763	0.869	0.59	− 5.77	− 4.35	− 3.44	− 2.10	√
THD13	0.674	0.371	0.603	0.868	0.63	− 6.39	− 5.68	− 4.69	− 2.02	×
THD14	1.16	0.508	0.658	0.869	0.53	− 5.20	− 3.43	− 2.35	0.16	√
THD15	0.835	0.668	0.824	0.866	0.96	− 4.17	− 3.26	− 2.16	0.09	√
THD16	1.059	0.492	0.739	0.867	0.75	− 3.77	− 3.03	− 2.41	− 0.43	√
THD17	1.066	0.581	0.816	0.866	1.00	− 3.41	− 2.12	− 1.20	0.34	√
THD18	1.008	0.468	0.733	0.864	1.25	− 2.96	− 1.90	− 1.00	0.48	√

‘√’ in the table is the selected item; ‘ × ’ to delete the item

### Validation of the CKD-PRO

There were 272 issued copies of the CKD-PROs in all, and 270 of them were retrieved for analysis.

### Reliability analysis

Cronbach’s *α* coefficients were calculated in four domains internally: 0.916 physiological, 0.893 psychological, 0.811 social domain, and 0.888 therapeutic. The coefficient for the entire scale was 0.939. The split-half reliability coefficient of the CKD-PROs was 0.945, and in the physiological, psychological, social, and therapeutic domains, it was 0.922, 0.904, 0.821 and 0.912, respectively. Thus, the scale showed excellent reliability.

### Content validity

The CVIs of all items were higher than 80%, indicating that there was acceptable content validity. In addition, in the preparation of the CKD-PROs scale, many relevant studies and domestic and foreign scales were consulted. Methods such as expert consultation and patient interviews were used to conduct in-depth and repeated argumentations on the optimization of the scale items to ensure that the scale had high content validity.

### Construct validity

**Fig. 3:**
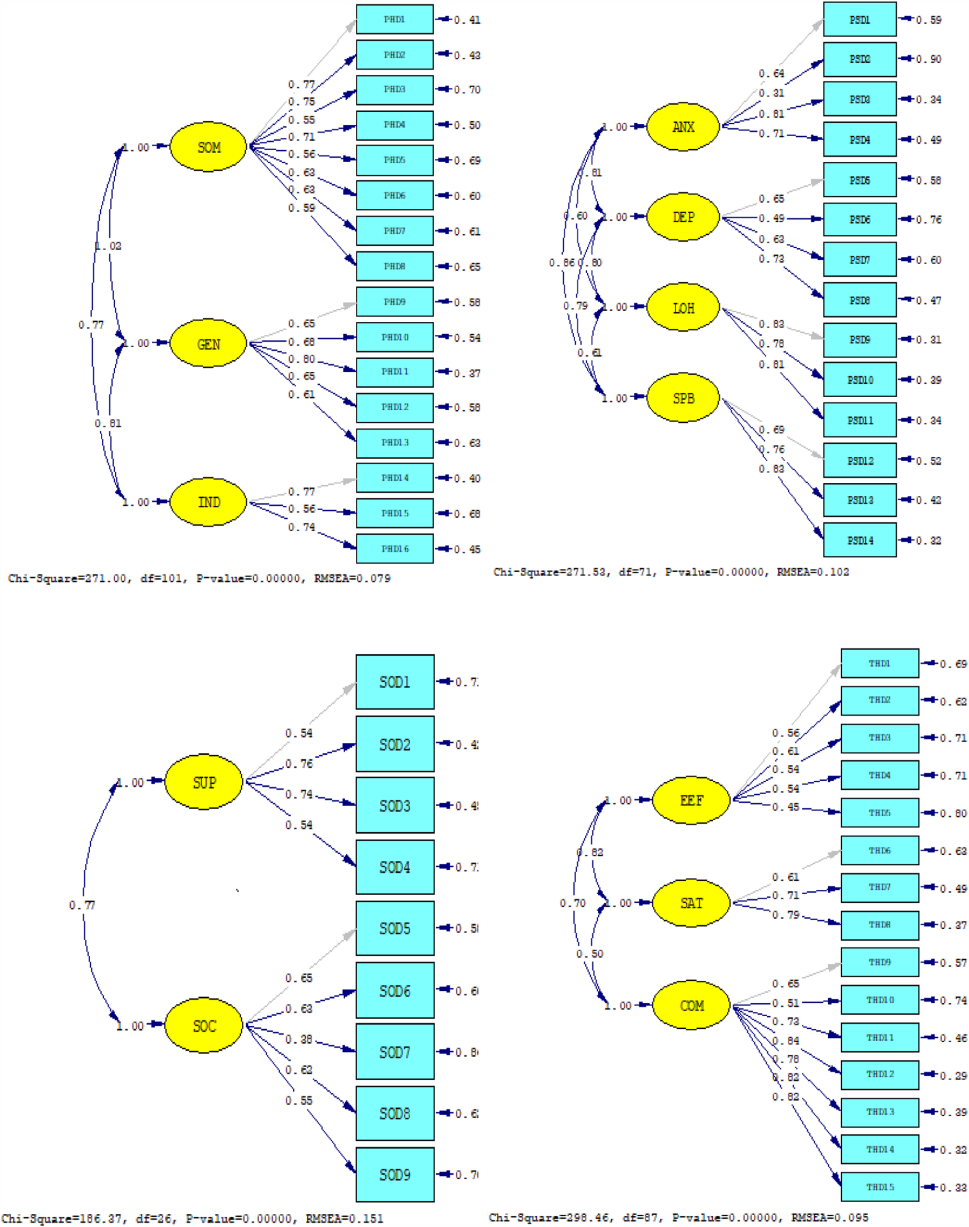
Confirmatory factor analysis model

The results show that the standard load solutions of each factor are all greater than 0.3. The results in Table 3 show that the values were all less than 8 except for the SOD field. The AGFI value of SOD was less than 0.8, but the AGFI value of other fields was greater than 0.8. Except for PSD, SOD RMSEA is greater than 0.1, SOD RMR is greater than 0.1, and all other fields are less than 0.1. The CFI value of the SOD field is 0.860, and the CFI value of other fields is greater than 0.9. The overall fitting of the model agrees with all the expressions, suggesting that the model has good structural validity (Fig. 3).

**Table 3:** Goodness-of-fit statistics of the CKD-PROs

Field	*X*^2^/df	AGFI	RMSEA	RMR	NFI	NNFI	IFI	CFI
PHD	2.683	0.850	0.079	0.061	0.950	0.960	0.970	0.970
PSD	3.824	0.810	0.100	0.091	0.930	0.930	0.950	0.950
SOD	7.168	0.770	0.150	0.140	0.840	0.800	0.860	0.860
THD	3.431	0.820	0.095	0.067	0.920	0.930	0.950	0.940
Total	1.740	0.850	0.052	0.051	0.970	0.980	0.990	0.990
standard	*X*^2^/df < 5 (8)	> 0.8	< 0.08 (0.1)	< 0.08	> 0.8	> 0.9 (0.8)	> 0.8	> 0.8

*X*^*2*^*/df* Chi-square value/degree of freedom, *AGFI* Adjust goodness-of-fit index, *RMSEA* root mean square of approximate error, *RMR* root mean square residual, *NFI* Specification fitting index, *NNFI* non-standard fitting index, *IFI* Value-added fitting index, *CFI* Comparative fitting index

### Dimensional correlation

There is a strong correlation between each item and its field, and the correlation number r of each item ranges from 0.413 to 0.669.

### Response analysis

In this survey, 2 measurements of 147 subjects before and after treatment were used, and the matched sample *t* test was used to analyze the 2 measurements. According to the results in Table 4, the scores of subjects before and after treatment were statistically significant except for GEN and DOS (all *p* < 0.05). The differences were all within a reasonable range, indicating that the scale can effectively distinguish patients before and after treatment and that the scale has a good response degree.

**Table 4:** The scores of all aspects of the scale were compared before and after treatment

Dimension	Score	*t*	*p*
SOM	− 0.06122 ± 0.15160	− 4.897	0.000
GEN	− 0.01905 ± 0.13515	− 1.709	0.090
IND	− 0.08390 ± 0.38194	− 2.663	0.009
ANX	− 0.11054 ± 0.28539	− 4.696	0.000
DEP	− 0.36565 ± 0.52119	− 8.506	0.000
LOH	− 0.11111 ± 0.31292	− 4.305	0.000
SPB	− 0.05442 ± 0.25891	− 2.549	0.012
SUP	− 0.12585 ± 0.38416	− 3.972	0.000
SOC	− 0.06259 ± 0.22306	− 3.402	0.001
EFF	− 0.10884 ± 0.37029	− 3.564	0.000
SAT	− 0.04762 ± 0.29967	− 1.927	0.056
COM	− 0.07289 ± 0.28833	− 3.065	0.003

### Feasibility analysis

A total of 636 questionnaires were issued in the 2 clinical investigations, and 630 questionnaires were finally collected with a recovery rate of 99.1%, among which 630 were effective for an effective rate of 100%. The completion time for each questionnaire was approximately 13 min. The above results show that this scale has good feasibility.

## Discussion

CKD progresses slowly and is irreversible. During the early stages of CKD, patients may not experience any obvious symptoms, making it difficult to monitor their condition. Self-report outcome measures can aid medical staff in monitoring the progression of the disease and determining if more frequent check-ups or treatment are necessary. As the patient enters stages 4–5 of CKD, the complexity of their condition increases and personalized treatment becomes necessary. Self-report outcome measures can help medical staff understand the patient’s experiences and problems, identify factors that may induce or worsen symptoms, assess the quality of life and progression of CKD, and develop targeted personalized treatment and management plans. In addition, the PROs can provide insight into the patient's needs and preferences when choosing kidney replacement therapy. For instance, by considering patient feedback and reports, the best treatment method for kidney transplant or dialysis can be determined. Therefore, the development of a new CKD-PROs measure can help medical staff better understand the patient’s condition and needs, thereby allowing them to develop better treatment plans and improve the patient’s quality of life.

In this research, we have described the development of a new method for measuring CKD-specific patient-reported outcomes. The CKD-PROs has made initial assessments of its reliability and validity. We hypothesized that the CKD-PROs can measure the symptoms and psychosocial impact of CKD. According to the guidance of the FDA on the development of patient-reported outcomes, the process has solicited and documented opinions from patients who met current consensus diagnostic guidelines of CKD in a broad base, including the four main clinical phenotypes CKDsPHD/CKDsPSD/CKDsSOD/CKDsTRE [25, 34, 35]. By doing so, it can ensure that their experience is accurately understood. We also conducted a draft instrument test made up of 79 items that were regarded as critical by patients in focus groups, which was later refined to 54 items based on an evaluation of their measurement properties in a CKD patient cohort.

With an increasing focus on patient-centered care, patient-reported outcome measurements (PROMs) allow clinicians and researchers to deliver healthcare services to patients more accurately. General health-related QoL measures (e.g., the SF-36, COSMIN) allow clinicians to compare the disease burden between chronic diseases. Disease-specific devices can capture specific CKD parameters that are clinically significant and are essential to clinical trials, which provide medical professionals with patients’ perspectives. According to the FDA PROMs guidelines for clinical trial endpoints, which were developed by patients with diseases and are under study, problems identified by PROMs must reflect patient progress and be corrected based on their input. A specific and appropriate recall period for the disease must be established, and data must demonstrate validity, reliability and responsiveness [25]. The PROMIS-57 and PROMIS-29 have been widely recognized as highly reliable and effective general tools for assessing patients’ disease experience in the field of CKD [36]. However, to our knowledge, there is no PROMs in CKD that fully meets the FDA criteria. Although CKD-PROs considers FDA acceptance as meeting the standard requirement, the PROMs was developed according to the prescribed methodology in the guidance statement.

Recently, we have become increasingly aware of the fact that it is important to obtain information from patients’ disease and treatment experience in regulatory and clinical fields [25, 37, 38]. The specific PROMs of CKD have some obvious characteristics in their development, such as variability and patient involvement. Many PROs measures have demonstrated limited psychometric effectiveness in CKD, and generic PROs measures assessing HRQOL or concept-specific outcomes have demonstrated limited content effectiveness in CKD. The generic HRQOL assessment questionnaire SF-36 shows good coverage of life impacts, but it may have limited value in the clinical trial setting due to other reasons. HRQOL outcomes may also be influenced by intervening factors over time. Disease-specific HRQOL measures, such as KDQOL-36 (which shows good conceptual coverage), encounter very similar challenges to generic HRQOL measures because the outcomes are closer to the disease or treatment, such as symptoms, are more likely to show a meaningful treatment and are more effective than downstream consequences, such as HRQOL. The latter is influenced by a range of factors.

The FDA issued guidelines on PROs research application and clinical drug development and efficacy evaluation, which defined PROs as any health status and treatment efficacy report directly from patients. PROs emphasizes the importance of patients’ subjective feelings and is a key indicator for disease treatments and treatment effects from the perspective of patients. The PROs scale can be used to measure a variety of patient factors (i.e., symptoms, psychological status, social participation, ability to perform daily living activities, and health-related quality of life) [18]. To ensure the rationality and objectivity of PROs evaluation, medical experts began to introduce psychological evaluation methods into PROs evaluation and developed many famous scales. One such scale, developed by the MAPI Institute in Lyon, is the PROs&QOLID database for patients’ Reported clinical outcome and quality of Life (PROs&QOLID), which sets up scale information in a structured form and provides extensive and in-depth information about PROs and quality of life for researchers engaged in health assessment through the network. The development of the PRO integrates the latest and most scientific research methods in measurement, investigation, health information technology, clinical research and qualitative research and establishes a set of health outcomes that can be used to measure the self-reported feeling, function, and status of multiple groups of people. The PROs development process typically involves the following several steps. First, field establishment and concept definition are undertaken, which involve reviewing the literature and collecting input from the patients, their family members, care providers, and clinical professionals and experts and the initial establishment of physiological function, fatigue, pain, emotional distress, social health, and overall health areas. Then, item pool formation and proofreading are conducted. The developer creates a list of potential items relevant to the concept of interest. These items can be general (e.g., “In general, would you say your health is: excellent, very good, good, fair, poor?”) or specific to a condition (e.g., “My kidney disease interferes too much with my life: strongly agree, agree, neither agree nor disagree, disagree, strongly disagree”). By adopting qualitative and quantitative research methods and by considering the existing filters, the items were classified, selected, evaluated, and modified according to whether the item was consistent with the defined field, the results of the item response theory analysis and the definition of the entries themselves. By considering these specific qualitative and quantitative aspects, it was determined whether to retain the item. Responses from focus groups and cognitive interviews were used to revise the reserved items, and finally, a pool of items in each field was formed.

The CKD-PROs is concise and accessible. It has the advantages of good reliability and validity and can also help medical staff assess specific symptoms, treatment preferences, and all kinds of aspects of overall health. CKD-PROs can incorporate the patient’s opinions and suggestions effectively into clinical care, clinical trials, and health care policies and eventually conduct customized high-quality care for patients with CKD. PROs assessment is highly recommended at every outpatient clinic follow-up. Future studies need to guarantee that more patients must be recruited, and they should be from multiple centers and even from different cultures and countries for the examination of the psychometric properties of the CKD-PROs. If you intend to use the scale developed in this study, please contact our team by email to obtain authorization for its use.

## Conclusion

In this study, researchers achieved complementary advantages by combining the CTT and IRT methods [39]. According to the results, item selection is of great significance in instrument development. First, we selected items considering their importance, suitability, and certainty by patient interviews and the Delphi method. Then, we analyzed the CTT methods, discrete trend method, correlation coefficient method, Cronbach’s alpha method, and other factor analysis methods from perspectives such as sensitivity, representativeness, independence, and internal consistency. The discrimination parameter (a), difficulty parameter (b), and item information were analyzed by the IRT method. The comprehensive application of these methods laid a good foundation for the screening of high-quality items. It may also present a scientific and effective approach for evaluating the clinical efficacy of CKD through PROs measures.

### Limitations

The current study has several limitations. First, the sample size of the survey is limited. Second, the questionnaire developed contains 54 items, which may be too large. In the next phase of our work, we plan to expand the sample size to enhance content validation and calibrate the item banks. In addition, we will employ CAT (computerized adaptive testing) technology to tailor the specificity of each item to a continuous range of a specific feature, such as the degree of skin itching. This approach promises to save assessment time and improve the efficiency of questionnaire completion.

## Data Availability

This article is allowed to be used, shared, adapted, distributed, and reproduced in any medium or format, provided that you identify the original author and source, provide a link to a Creative Commons license, and indicate whether modifications have been made.
